# The usual SASPects of liver cancer

**DOI:** 10.18632/aging.100758

**Published:** 2015-06-02

**Authors:** Amaia Lujambio, Augusto Villanueva

**Affiliations:** Division of Liver Diseases Icahn School of Medicine at Mount Sinai, New York, NY 10029

Cellular senescence is a stable form of cell cycle arrest that limits the propagation of damaged cells and can be triggered in response to diverse forms of cellular stress [1]. This anti-proliferative program is mediated by the p53 and Rb pathways and was initially considered a cell-autonomous mechanism that promotes tumor suppression and tissue homeostasis [1]. However, several groundbreaking studies performed in the last decade have established that senescent cells can impact their environment through the secretion of growth factors, cytokines, chemokines, immune modulators and extracellular matrix-degrading enzymes [1]. This process, collectively known as the senescence-associated secretory phenotype (SASP), enables the non-cell-autonomous activities of senescent cells. The functions exerted by the SASP are diverse and include the autocrine reinforcement of cell cycle arrest as well as the paracrine transmission of the senescent phenotype to neighboring cells, thereby maintaining and propagating tumor suppression [1]. Moreover, SASP can directly modulate the tissue microenvironment, elicit immune surveillance of senescent cells, and paradoxically, promote tumorigenesis by supporting the proliferation of surrounding malignant or pre-malignant cells [1].

Many of the findings that illustrate the impact of SASP on the microenvironment stem from in vivo studies in the liver. This vital organ executes complex functions that include metabolizing environmental toxins, drugs, and alcohol. The excessive exposure to these harmful chemicals or infection with hepatitis B and C viruses induce liver injury, which disrupts liver architecture and frequently results in hepatocellular carcinoma (HCC), a very aggressive type of liver cancer that arises in a damaged environment. Liver cancer is currently the second cause of cancer-related death worldwide, with a mortality rate that has increased more than 50% in the last 2 decades. A seminal study demonstrated that the cellular senescence program can engage the immune system to produce complete tumor regressions [2]. By using an Hras-driven mouse liver cancer model in which the endogenous levels of p53 can be modulated by inducible RNA interference, reactivation of p53 expression led to cellular senescence and the generation of a SASP that triggered the infiltration of natural killer cells and other innate effector cells to eliminate senescent tumor cells. More recent data showed that oncogenic Nras-induced hepatocyte senescence followed by senescence surveillance acts as an efficient barrier to liver tumorigenesis [3]. Both the innate and the adaptive immune systems were involved in the elimination of pre-malignant senescent hepatocytes, suggesting that different senescence triggers could provoke diverse immune responses.

Cellular senescence can also be triggered in the stromal compartment of the liver. Upon liver injury, hepatic stellate cells (HSCs) activate, proliferate, and develop a profibrotic secretome. Activated HSCs eventually undergo cellular senescence and produce a SASP enriched in fibrolytic molecules, contributing to fibrosis resolution [4]. Moreover, senescent HSCs also secrete pro-inflammatory cytokines that direct the immune surveillance of senescent HSCs, further limiting liver fibrosis. In a following study, mice were subjected to increasing levels of liver damage and p53-mediated senescence of HSCs was reported to restrict liver cirrhosis and HCC development [5]. Senescent HSCs produced a SASP that favored M1 polarization of the macrophages, thereby creating an anti-tumor microenvironment. In contrast, proliferating HSCs secreted molecules that switched macrophage polarization into the M2-type, contributing to a protumorigenic milieu.

The production of a proper SASP and subsequent immune-mediated clearance of senescent cells appear to be critical for the beneficial effects of cellular senescence on liver homeostasis and tumor suppression. Accordingly, genetic or chemical abrogation of the immune system leads to increased liver fibrosis, liver cancer, and delayed tumor regression after p53 reactivation in liver cancer cells [2-4]. Intriguingly, in a murine model of HCC driven by a chemical carcinogen and obesity, senescence of HSCs and the corresponding SASP were associated with hepatocarcinogenesis [6]. These contradictory findings could potentially be explained by differences in the senescence trigger, in the composition of the SASP, or by defective senescence surveillance. In fact, the clearance of senescent HSCs was not observed in the latter study [6], further emphasizing the importance of efficiently eliminating senescent cells.

Pro-senescence therapy has recently emerged as a novel therapeutic approach for treating cancer and could be applied to liver cancer, a disease that lacks effective treatment. However, if senescent tumor cells are not properly eliminated by the immune system, the SASP can promote the growth of non-senescent adjacent tumor cells. One solution could be to manipulate SASP to restrict its protumorigenic properties and/or enhance its ability to engage the immune system. An elegant work clearly showed how Pten-loss-induced senescence creates an immunosuppressive and protumorigenic microenvironment in prostatic intraepithelial neoplasias [7]. However, pharmacological inhibition of the Jak2/Stat3 pathway reprogrammed SASP, restoring immune surveillance and the anti-tumor effects. Another appealing option is to boost the immune system to improve the surveillance of senescent tumor cells. Treatment with the antiprogrammed cell death protein 1 (PD1) immune checkpoint antibodies or ipilimumab, an antibody that enhances the activation of cytotoxic T cells by blockade of the cytotoxic T-lymphocyte associated protein 4 (CTLA-4) receptor, could improve the anti-tumor potential of pro-senescence therapies. Understanding and manipulating the signaling pathways that control SASP as well as identifying the key mediators of SASP will be essential to unleash the full potential of the senescence program.
